# Transcriptome analyses reveal molecular mechanisms of novel compound heterozygous *ACO2* variants causing infantile cerebellar retinal degeneration

**DOI:** 10.3389/fncel.2024.1492048

**Published:** 2024-11-12

**Authors:** Wenke Yang, Shuyue Wang, Ke Yang, Yanjun Li, Zhenglong Guo, Jianmei Huang, Jinming Wang, Shixiu Liao

**Affiliations:** ^1^Henan Provincial People's Hospital, People's Hospital of Henan University, People's Hospital of Zhengzhou University, Zhengzhou, China; ^2^National Health Commission Key Laboratory of Birth Defects Prevention, Henan Provincial Key Laboratory of Genetic Diseases and Functional Genomics, Zhengzhou, China; ^3^Department of Gynaecology and Obstetrics, Central Hospital of Wuhan, Wuhan, China

**Keywords:** ACO2, variant, infantile cerebellar retinal degeneration, neuropathy, mitochondrial aconitase activity

## Abstract

**Background and purpose:**

Infantile cerebellar retinal degeneration (ICRD) (OMIM #614559) is a rare autosomal recessive inherited disease associated with mutations in the aconitase 2 (ACO2) gene. We report a Chinese girl with novel compound heterozygous variants in *ACO2*, who presented at 7 months of age with psychomotor retardation, truncal hypotonia, and ophthalmologic abnormalities. This study aims to investigate the potential molecular mechanisms underlying *ACO2* deficiency-induced neuropathy.

**Methods:**

Whole exome sequencing was performed on family members to screen for potential pathogenic mutations, followed by Sanger sequencing for validation. Mitochondrial aconitase activity and mitochondrial DNA (mtDNA) copy number were measured using an aconitase activity detection kit and quantitative PCR, respectively. Transcriptome expression profiles from patient cells, and cerebellar and retinal organoids retrieved from the GEO database were integrated. Functional enrichment analysis and protein-protein interaction networks were used to identify key molecules, and their expression levels were validated using Western blot analysis.

**Results:**

Genetic testing revealed novel compound heterozygous variations in the proband's *ACO2* gene (NM:001098), including c.854A>G (p.Asn285Ser) and c.1183C>T (p.Arg395Cys). Predictive analysis of the tertiary structure of the ACO2 protein suggests that both p.Asn285Ser and p.Arg395Cys affect the binding ability of ACO2 to ligands. The mitochondrial aconitase activity and mtDNA copy number in the proband's leukocytes were significantly reduced. Transcriptomic data analysis identified 80 key candidate genes involved in ACO2-related neuropathy. Among these, *LRP8* and *ANK3*, whose gene expression levels were significantly positively correlated with *ACO2*, were further validated by Western blot analysis.

**Conclusions:**

This study expands the spectrum of pathogenic *ACO2* variants, elucidates the potential molecular mechanisms underlying ACO2-related neuropathy, provides in-depth support for the pathogenicity of *ACO2* genetic variations, and offers new insights into the pathogenesis of ICRD.

## Introduction

The *ACO2* gene, located on chromosome 22q13.2, spans approximately 59.9 kb and consists of 18 exons. The encoded ACO2 protein is a monomeric mitochondrial enzyme with 780 amino acids and a molecular weight of approximately 85 kDa. It belongs to the aconitase isomerase family and contains four iron-sulfur cluster binding sites essential for its catalytic activity (Gruer et al., 1997). ACO2 catalyzes the conversion of citrate to isocitrate via cis-aconitate, the second step in the tricarboxylic acid (TCA) cycle, also known as the Krebs cycle (Jung et al., 2015). Some studies suggest ACO2 also affects mitochondrial DNA (mtDNA) maintenance, further contributing to mitochondrial function (Sadat et al., 2016; Neumann et al., 2020; Lail et al., 2023). Impaired ACO2 activity has been implicated in immunity and neurodegenerative diseases (Kim et al., 2023; Gille and Reichmann, 2011; Zhu et al., 2023). However, the molecular mediators for ACO2-related phenotypic diversity remain largely unknown.

Pathogenic variants in the *ACO2* gene can lead to a wide clinical spectrum of neuropathy. The severity and specific symptoms depend on whether monoallelic or biallelic mutations are affected and the impact of the mutation on enzyme activity. Pathogenic *ACO2* mutations cause dominant optic atrophy, characterized by progressive vision loss due to optic nerve degeneration (Neumann et al., 2020; Cerrada et al., 2019; Charif et al., 2021). These mutations typically result in a 50%−60% reduction in ACO2 activity, leading to mitochondrial dysfunction and retinal ganglion cell death (Sharkia et al., 2019). The rare autosomal recessive Infantile Cerebellar-Retinal Degeneration (ICRD) disorder is caused by homozygous or compound heterozygous mutations in *ACO2* (Spiegel et al., 2012; Blackburn et al., 2020). ICRD manifests with severe neurological problems like developmental delay, intellectual disability, hypotonia, spastic paraplegia, optic atrophy, and retinal degeneration (Spiegel et al., 2012; Blackburn et al., 2020). These mutations significantly disrupt ACO2 function, causing below 35% of normal enzyme activity and leading to the accumulation of toxic metabolites that damage the cerebellum and retina (Spiegel et al., 2012; Abela et al., 2017; Metodiev et al., 2014). Other homozygous or compound heterozygous *ACO2* mutations causing about 50% of normal enzyme activity have been linked to a spectrum of phenotypes, including complex spastic paraplegia complicated by intellectual disability and microcephaly (Bouwkamp et al., 2018), complex spastic paraplegia with episodic visual loss (Tozawa et al., 2021), and recessive optic atrophy with or without spastic paraplegia (Marelli et al., 2018; Gibson et al., 2020). These cases often show higher residual ACO2 activity compared to ICRD. Previous studies have also evaluated the effects of *ACO2* mutations on mitochondrial respiratory chain and TCA metabolites (Sadat et al., 2016; Abela et al., 2017; Bouwkamp et al., 2018). However, the molecular mechanisms from mitochondrial dysfunction caused by impaired ACO2 activity to generalized neuropathy have not been elucidated. Therefore, it is necessary to explore the development mechanism of neuropathy in a meaningful model.

In this study, we report on novel compound heterozygous *ACO2* variants identified in an infant with psychomotor retardation, truncal hypotonia, and ophthalmologic abnormalities. We used peripheral leukocytes obtained from family members to characterize enzyme activity, mtDNA copy number, and transcriptome expression profile. Leukocytes with biallelic *ACO2* mutations showed decreased mitochondrial ACO enzyme activity and reduced mtDNA copy number. The transcriptome profiling from leukocytes and the transcriptome datasets of cerebellar and retinal organoids retrieved from the GEO database were integrated and analyzed to reveal the key molecules involved in *ACO2* pathogenic variants causing neuropathies. Our study expands the pathogenic mutation spectrum of *ACO2*, and transcriptomic results provide valuable clues for understanding the molecular mechanisms of ACO2-related disorders, which may help reveal pathological progression, discover new biomarkers, and develop new treatments.

## Methods

### Patient and ethics

**Figure 1:**
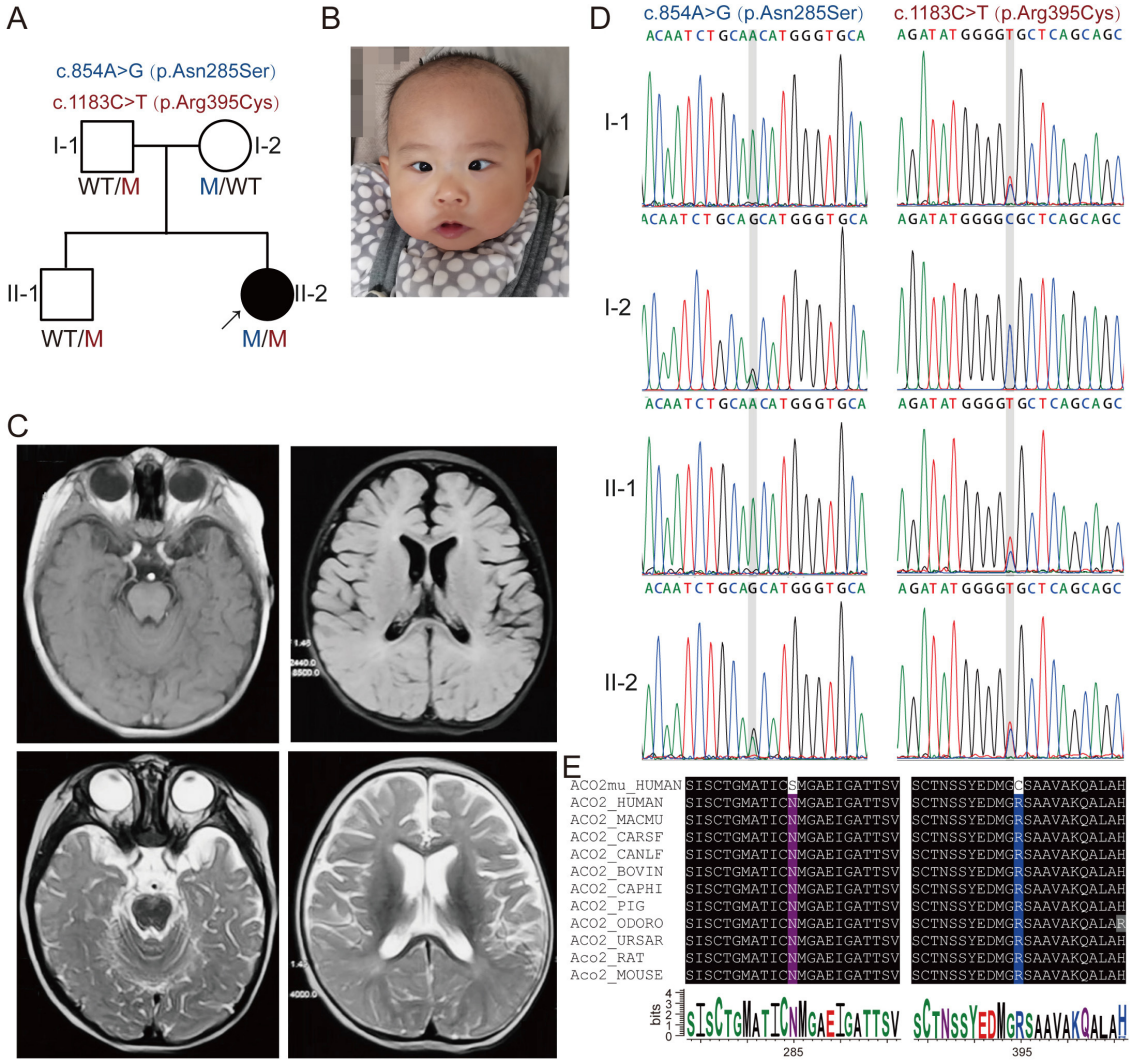
Segregation of novel compound heterozygous *ACO2* variants in a family with neuropathic phenotypes. **(A)** Family pedigree of the patient with novel compound heterozygous *ACO2* variants c.854A>G (p.Asn285Ser) and c.1183C>T (p.Arg395Cys). The proband is indicated with an arrow, affected family members with black symbols, and unaffected members with white symbols. Men are represented by squares, and women by circles. **(B)** Facial feature of the proband (II-2). **(C)** Representative brain magnetic resonance imaging of the proband (II-2). **(D)** Representative chromatograms for c.854A>G and c.1183C>T compound heterozygous mutations of *ACO2* in the family, indicated by gray shades. **(E)** The affected amino acid residues in ACO2 are highly conserved across 11 species.

We ascertained a non-consanguineous Chinese family (Figure 1A) with a 14-month-old female proband exhibiting global developmental delay and esotropia. This family has no affected relatives in previous generations. The study was approved by the Ethics Committee of Henan Provincial People's Hospital (approval number: 2019_134) in accordance with the Declaration of Helsinki. Written informed consent was obtained from adult participants or the guardians of minor participants.

### Whole-exome sequencing and bioinformatics analysis

Whole-exome sequencing (WES) of four family members from the first and second generation was performed as previously described. After quality assessment using FastQC (https://www.bioinformatics.babraham.ac.uk/projects/fastqc/), the raw sequencing data were aligned to the reference human genome build hg19 using BWA software (https://sourceforge.net/projects/bio-bwa/). Picard software (http://broadinstitute.github.io/picard) was used for statistical analysis of the aligned results and to remove PCR amplification duplicates. After correction using the Genome Analysis Toolkit (GATK) standard process, GATK HaplotypeCaller (https://software.broadinstitute.org/gatk/) was used to identify single nucleotide variants and InDels in each sample. ANNOVAR (http://www.openbioinformatics.org/annovar/) was then used to annotate the variants. Variants related to the neurophysiology were considered as candidate variants. Sanger sequencing was performed on all available DNA samples from the enrolled family to confirm the genotype, and co-segregation analysis was carried out to identify causal variants. Evolutionary conservation of the identified causal variants across species was explored using the reference sequences released from NCBI (https://www.ncbi.nlm.nih.gov/gene) and UniProt (https://www.uniprot.org/) databases. SwissModel server (http://swissmodel.expasy.org/), LigPlot+ software (http://www.ebi.ac.uk/thornton-srv/software/LIGPLOT/), MetaDome (https://stuart.radboudumc.nl/metadome/), mCSM-Stability (https://biosig.lab.uq.edu.au/mcsm/stability), and DynaMut2 (https://biosig.lab.uq.edu.au/dynamut2/) were used to predict the effect of the identified mutations on protein properties.

### Mitochondrial aconitase activity assay

Peripheral blood samples were collected, followed by red blood cell lysis (Solarbio, Beijing, China) to obtain peripheral blood leukocytes. According to the instructions of the Aconitase Assay Kit (Abbkine, Wuhan, China), 10 × 10^6^ cells were harvested, and a precipitate containing mitochondrial fractions was obtained after homogenization and centrifugation. The precipitate was resuspended in lysis buffer and then sonicated using a Bioruptor sonication water bath (Diagenode). The mixture was centrifuged at 5,000 × *g* for 2 min, and the supernatant was transferred to a microplate. The absorbance change after 5 min of reaction with Working Solution was detected at 340 nm, and these values were used to calculate the activity of mitochondria aconitase.

### Relative mitochondrial DNA copy number

Genomic DNA was isolated from the peripheral blood leukocytes. After extraction, the DNA concentration and purity were measured using a Nanodrop2000. A total of 1 μg DNA per sample was prepared for quantitative real-time PCR (qRT-PCR) assays using primer pairs targeted to mitochondrial gene mtND1 or nuclear gene β-Actin. qRT-PCR was performed with Taq Pro Universal SYBR qPCR Master Mix (Vazyme, Nanjing, China) using a StepOnePlus Real-Time PCR System (Applied Biosystems). The ratio of mitochondrial DNA to nuclear DNA, which determined the relative copy number of mitochondrial DNA, was calculated using the 2^−(Δ*ΔCt*)^ method.

### RNA sequencing analysis and differential gene set analysis

Total RNA was isolated from the peripheral blood leukocytes using TRIzol reagent (Life Technologies, Carlsbad, CA). RNA sequencing was performed by Genesky Biotechnologies Inc. (GeneSky, Shanghai, China). Briefly, oligo-dT magnetic beads were used to separate mRNA from total RNA for construction of cDNA libraries. RNA-seq was performed on Illumina HiSeq. 2000 platform. Raw sequencing reads were evaluated by FastQC (https://www.bioinformatics.babraham.ac.uk/projects/fastqc/), then trimmed by Cutadapt (https://github.com/marcelm/cutadapt) to remove the adaptors and low-quality sequences. Clean reads were aligned to reference human genome GRCh38. Differentially expressed genes (fold change ≥2 and *P* < 0.05) were determined using edgeR (https://bioconductor.org/packages/edgeR/) and subjected to Gene Set Enrichment Analysis (GSEA) for Gene Ontology (GO) and Kyoto Encyclopedia of Genes and Genomes (KEGG) pathway enrichment in clusterProfiler package (https://bioconductor.org/packages/clusterProfiler/). Furthermore, we also conducted Gene Set Variation Analysis (GSVA) using GSVA package (https://bioconductor.org/packages/GSVA/) in R software.

### Data sources, gene co-expression analysis and protein-protein interaction

Given that individuals with loss-of-function mutations in the *ACO2* gene exhibit cerebellar and retinal organ damage, cerebellar and retinal organoids offer a valuable *in vitro* model for investigating the effects of *ACO2* mutations on neuronal development, function, and gene expression. To this end, we obtained gene expression profiles of cerebellar and retinal organoids (GSE161549 and GSE254830) from the Gene Expression Omnibus (GEO, http://www.ncbi.nlm.nih.gov/geo). The GSE161549 (Silva et al., 2021) and GSE254830 datasets included 15 and 12 samples, respectively. From the combined 27 samples, we calculated a ranked gene list based on the Pearson's correlation of each gene with *ACO2* expression, and this pre-ranked gene list was then loaded for GSEA. Additionally, weighted gene co-expression network analysis (WGCNA) was performed to construct co-expression modules based on the transcriptome data of cerebellar and retinal organoids. The R package clusterProfiler was used to identify the GO terms significantly enriched in co-expression modules containing the *ACO2* gene. Furthermore, genes selected from neurophysiological-related pathways were considered as key molecules. The STRING online database (https://string-db.org) was used to predict protein-protein interaction (PPI) networks of the key molecules.

### Western blot analysis

Freshly prepared lysates from leukocytes were used for Western blot analysis. Equivalent amounts of protein were separated by 10% SDS–polyacrylamide gel electrophoresis and then transferred to PVDF membranes (Millipore, Massachusetts, USA). The membranes were blocked for 1 hour at room temperature with 5% nonfat milk in TBS-Tween solution and then incubated with primary antibodies against ACO2 (ab129069, abcam), LRP8/APOER2 (ab108208, abcam), ANK3 (ab306589, abcam), and β-Actin (AF7018, Affinity Biosciences) at 4°C overnight. Subsequently, the membranes were washed three times for 10 min with TBS-Tween, and incubated with horseradish peroxidase–conjugated goat anti-rabbit IgG secondary antibody (S0001, Affinity Biosciences) for 1 h at room temperature. The blots were imaged with chemiluminescence reagents (34096, Thermo Fisher Scientific) and the ChemiDoc Imaging System (BioRad). The bands were quantified using ImageJ software (NIH, Bethesda, MD, USA).

### Statistical analysis

All statistical analyses were performed with an unpaired *t*-test or one-way ANOVA. Data were presented as means ± standard derivations, and *P* < 0.05 was considered statistically significant.

## Results

### Clinical characteristics of the proband

The proband (II-2) was a 14-month-old Chinese girl (Figure 1A), born at full term with a birth weight of 2.9 kg. A neurodevelopmental examination at 7 months of age, performed according to the Gesell Development Schedules, showed significantly lower scores in gross motor, fine motor, adaptability, language, and social-emotional response (35, 29, 44, 68, and 58, respectively) compared to healthy children of the same age. She exhibited truncal hypotonia, was incapable of rolling over, and had esotropia with no eye contact (Figure 1B). The Babinski sign was negative bilaterally. Flash electroretinogram responses showed markedly reduced amplitudes for both a- and b-waves. Cranial magnetic resonance imaging revealed vertical tortuosity of the right optic nerve and bilateral frontotemporal atrophy with widening and deepening of the sulci (Figure 1C).

### Genetic analysis identified novel compound heterozygous *ACO2* variants in the patient

**Figure 2:**
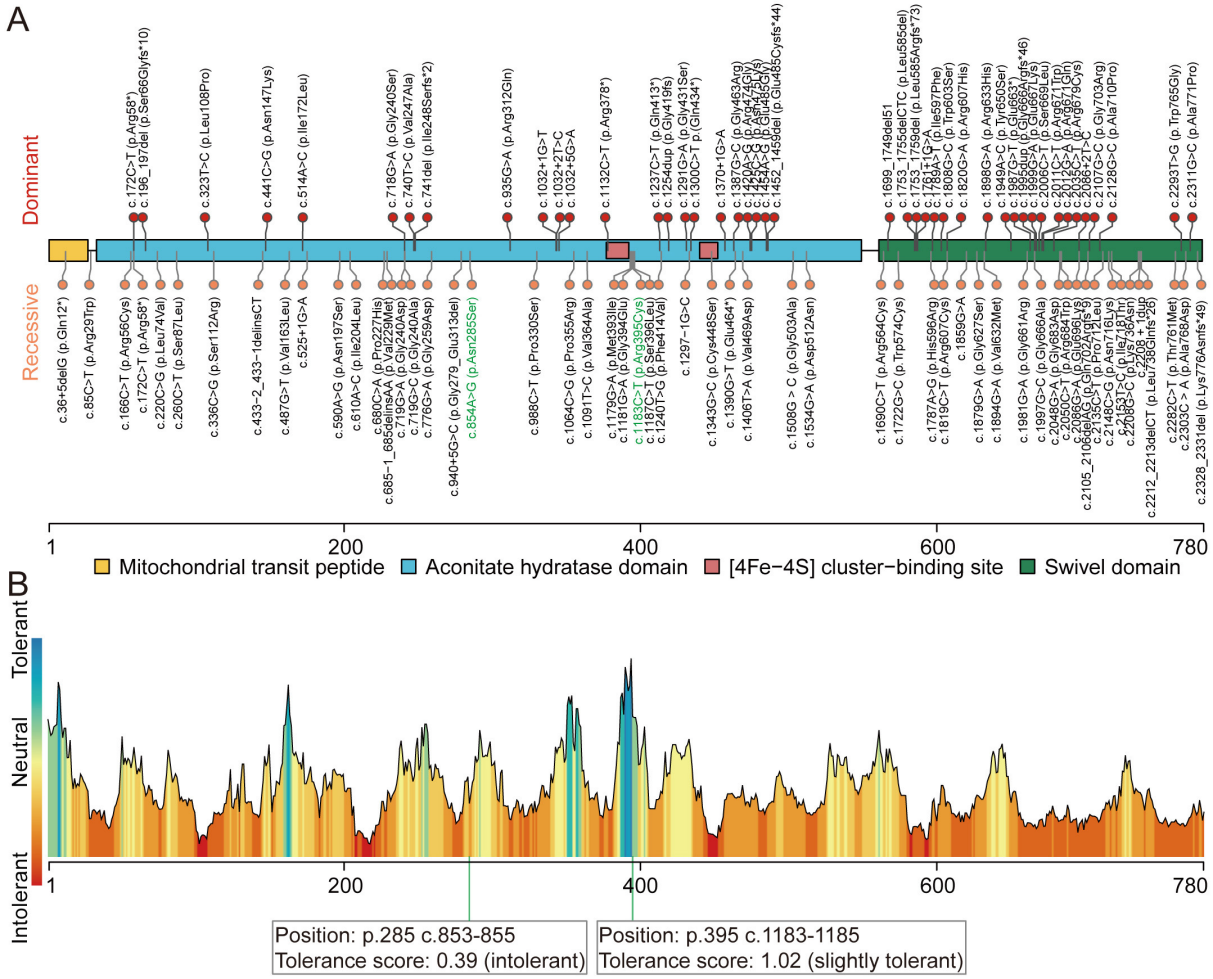
Pathogenic variants in *ACO2*. **(A)** Schematic of ACO2 (UniProt: Q99798) domain composition, annotated with the position of known and novel pathogenic variants. Protein domains are represented by different colored areas. The identified dominant variants (upper) are associated with dominant optic atrophy. The identified recessive variants (lower) are associated with phenotypes including ICRD, recessive optic atrophy with or without spastic paraplegia, and complex spastic paraplegia complicated by intellectual disability and microcephaly. Variants identified in this study are written with green letters. **(B)** Tolerance landscape visualization for missense variants in ACO2 via MetaDome. The p.Asn285 position is “intolerant,” while the p.Arg395 position is “slightly tolerant” for predicted amino acid substitutions.

Whole-exome sequencing (WES) was performed on DNA from the proband (II-2) and three healthy family members (I-1, I-2, and II-1; Figure 1A). This analysis identified novel compound heterozygous variants c.854A>G (p.Asn285Ser) and c.1183C>T (p.Arg395Cys) in *ACO2* (NM_001098) with strongly overlapping phenotypes in the study family. Sanger sequencing and segregation analysis confirmed that the proband (II-2) carried the compound heterozygous variants, with the paternally inherited c.1183C>T (p.Arg395Cys) and maternally inherited c.854A>G (p.Asn285Ser; Figure 1D). Homological comparisons suggested that the altered amino acid residues were highly conserved across species (Figure 1E). Although the paternally inherited variant c.1183C>T (p.Arg395Cys) was recorded in ClinVar (Variation ID: 1016737) and classified as of uncertain significance, it had not been previously reported in the literature. The maternally inherited variant c.854A>G (p.Asn285Ser) had not been reported before in the literature or variant databases (Figure 2A, Table 1). Genetic tolerance for each amino acid position in ACO2 (UniProt: Q99798) was further predicted by MetaDome. The p.Asn285Ser variant was considered “intolerant” (tolerance score = 0.39), while the p.Arg395Cys variant was considered “slightly tolerant” (tolerance score = 1.02; Figure 2B). Multiple *in silico* prediction algorithms, including SIFT, Polyphen2, MutationTaster, MutationAssessor, and PROVEAN, predicted both variants to be pathogenic (Table 1). Furthermore, using mCSM-Stability and DynaMut2, protein stability changes upon mutations were calculated. The p.Asn285Ser variant was predicted to be destabilizing (ΔΔ*G* = −0.295 kcal/mol) according to mCSM-Stability but stabilizing (ΔΔ*G* = 0.14 kcal/mol) via DynaMut2. Both stability predictors evaluated p.Arg395Cys as destabilizing (Table 1). Taken together, our study reveals that the *ACO2* missense compound heterozygous variants c.854A>G (p.Asn285Ser) and c.1183C>T (p.Arg395Cys) might cause ICRD in the studied family.

**Table 1:** Characteristics of *ACO2* (NM_001098) variants and the prediction of pathogenicity by different *in silico* tools.

**Variation (hg19)**	**ClinVar**	**HGMD**	**ACMG**	**gnomAD**	**SIFT**	**Polyphen2**	**Mutation taster**	**Mutation assessor**	**PROVEAN**	**CADD**	**mCSM-Stability (*ΔΔG*)**	**DynaMut2 (*ΔΔG*)**
chr22:41913549; c.854A>G; p.Asn285Ser	–	–	VUS (PM2, PP3)	–	Deleterious (0)	Probably damaging (1)	Disease_causing (1)	High functional impact (4.71)	Deleterious (−4.93)	27.3	−0.295 kcal/mol	0.14 kcal/mol
chr22:41918878; c.1183C>T; p.Arg395Cys	Uncertain significance	–	VUS (PM2, PP3)	0.00001	Deleterious (0)	Probably damaging (1)	Disease_causing (1)	High functional impact (3.535)	Deleterious (−7.97)	32	−1.924 kcal/mol	−1.09 kcal/mol

HGMD, Human Gene Mutation Database; ACMG, American College of Medical Genetics and Genomics; VUS, variant of unknown significance; gnomAD, genome aggregation database; CADD, Combined Annotation Dependent Depletion.

### Patient with compound heterozygous variants in *ACO2* presents with reduced mitochondrial DNA levels and ACO2 enzyme activity

**Figure 3:**
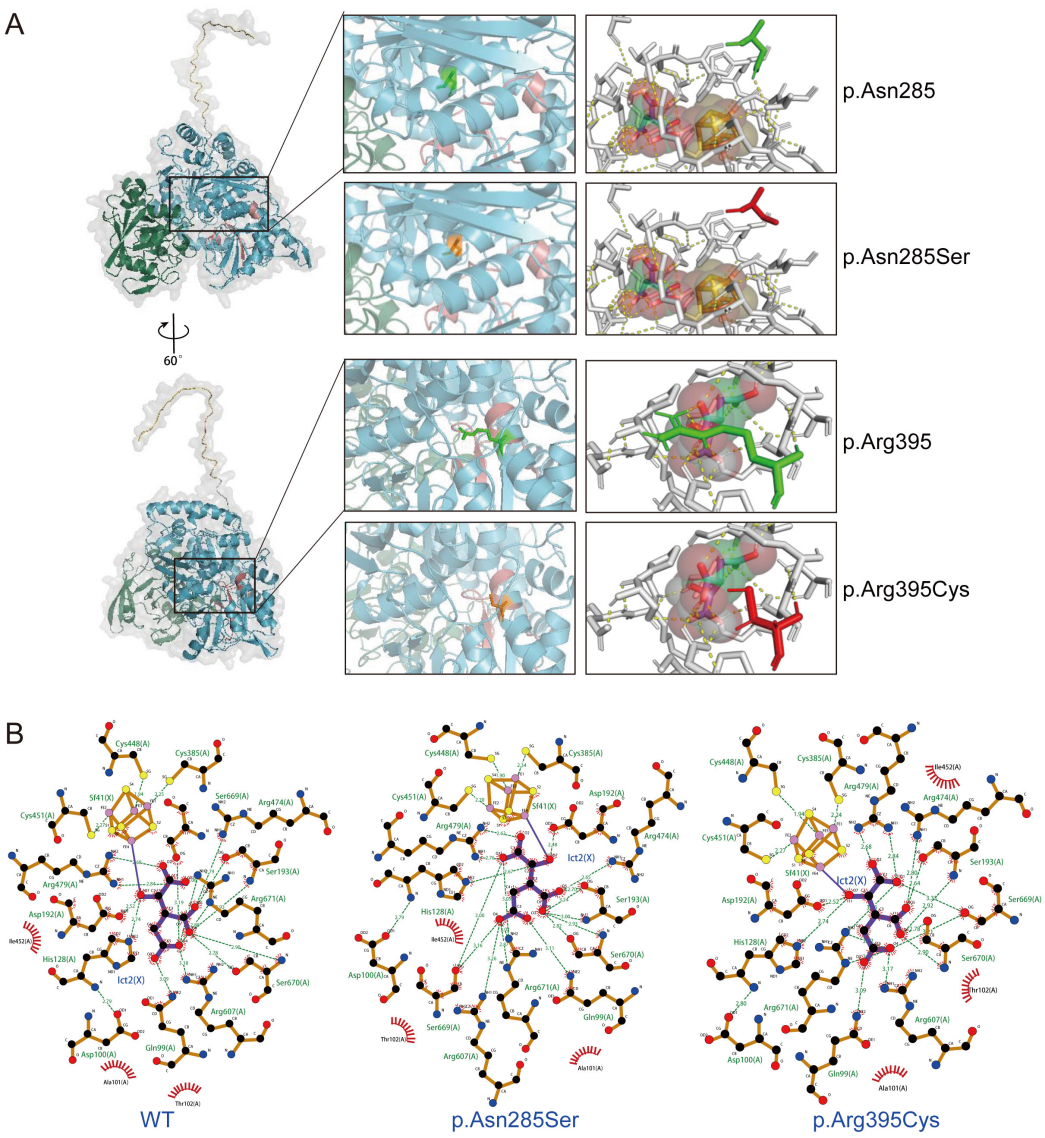
Visualization of the molecular docking of ACO2 with iron–sulfur cluster and isocitrate ligands. **(A)** Molecular docking results showing the optimal binding models of wild-type and variants (p.Asn285Ser and p.Arg395Cys) of ACO2 in complex with iron–sulfur cluster and isocitrate ligands. Prediction of the tertiary structures of ACO2 protein. Different colors indicate different functional domains **(left)**. The wild-type residues are presented as green sticks and the mutant residues are shown in orange sticks **(middle)**. The orange-yellow spheres represent iron–sulfur cluster. The red-green-white spheres represent isocitrate. Yellow dashed lines represent hydrogen bonds **(right)**. **(B)** LigPlot images show the amino acid residues around the ligands binding pocket with hydrogen bond networks and hydrophobic interactions. Sf41(X) denotes iron–sulfur cluster. Ict2(X) denotes isocitrate. Green dashed lines represent hydrogen bonds. The numbers denote the distances in angstrom unit. The sunburst icons around atoms represent hydrophobic interactions.

Figure 2A illustrates the domain of the ACO2 protein and the variants reported in ACO2-related disorders, including those identified in this study. Previous findings indicated a lack of hotspot *ACO2* variants, with the phenotypic heterogeneity of ACO2-related disorders depending on residual aconitase enzymatic activity rather than variant genotypes. Here, molecular docking was used to predict the binding models of wild-type and variant ACO2 (p.Asn285Ser and p.Arg395Cys) in complex with iron–sulfur cluster and isocitrate ligands. The predictions indicated that the p.Asn285Ser and p.Arg395Cys variants disrupted hydrogen bonding with neighboring amino acid residues observed in the wild-type protein (Figure 3A). Additionally, the arrangement of amino acid residues surrounding the ligand-binding pocket revealed alterations in the hydrogen bond network and hydrophobic interactions in the variants compared to the wild type (Figure 3B). These potential conformational changes may destabilize the interaction between ACO2 and its ligands by affecting binding energy, ultimately leading to impaired mitochondrial ACO activity.

**Figure 4:**
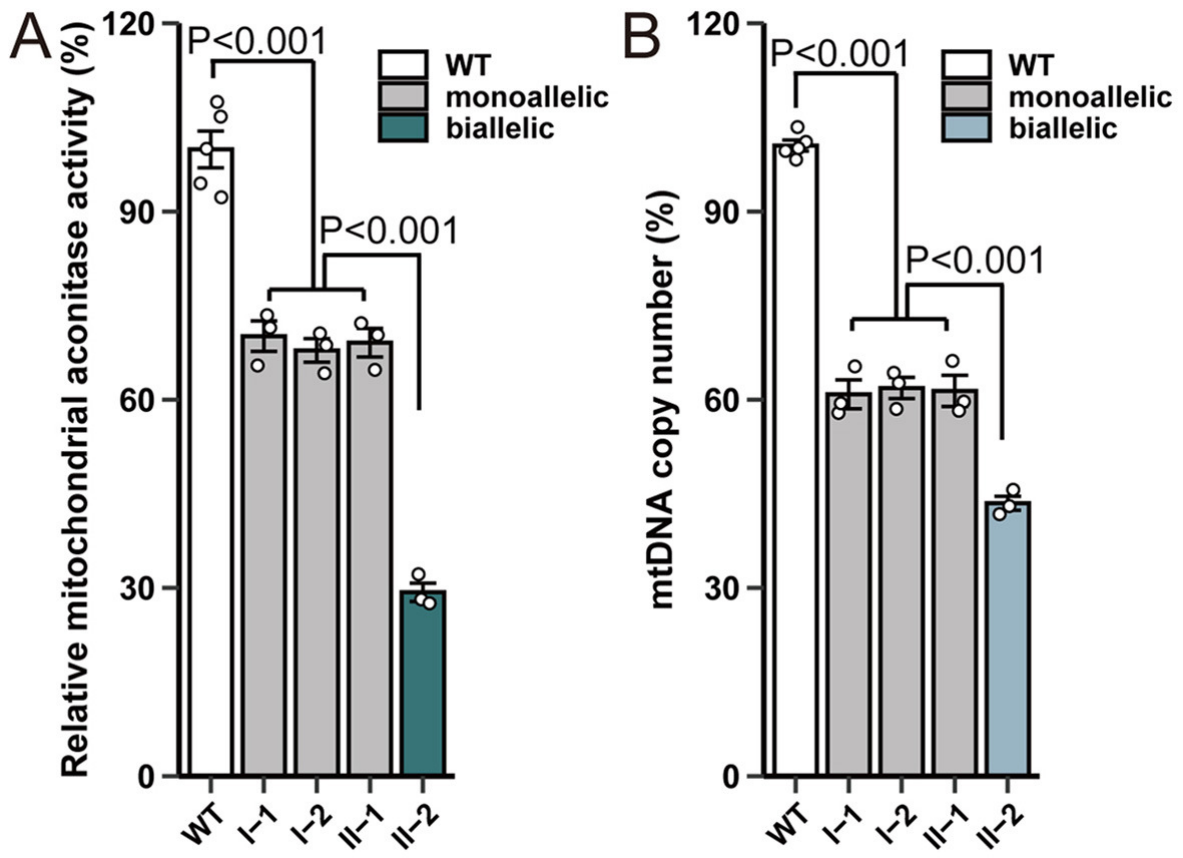
*ACO2* variants show impaired enzyme activity and mtDNA copy number. **(A)** Effect of variants on mitochondrial aconitase activity. The enzymatic activity was normalized based on the protein concentration of the cell lysates. The enzymatic activity in cells harboring wild-type *ACO2* was designated as 100%. Plotted values represent the means of technical replicates (mean ± SD). Statistical significance between groups was determined using one-way ANOVA with Tukey's multiple comparisons test. **(B)** Effect of *ACO2* variants on mtDNA copy number. Plotted values represent the means of technical replicates (mean ± SD). Statistical significance between groups was determined using one-way ANOVA with Tukey's multiple comparisons test.

Next, aconitase enzyme activity was measured using mitochondrial extracts from peripheral blood leukocytes of five individuals with wild-type *ACO2* and four family members. Our results showed that in the case of biallelic *ACO2* variants, mitochondrial aconitase activity was approximately 29.3% of that in *ACO2* wild-type cells (Figure 4A). In the case of monoallelic *ACO2* variant, mitochondrial aconitase activity remained at 69.0% (Figure 4A).

RT-PCR was used to further evaluate mtDNA levels in ACO2-mutated leukocytes. Compared with control cells, the mtDNA copy number in mitochondria of biallelic *ACO2* variants decreased to 43.5%. The mtDNA copy number in mitochondria of monoallelic *ACO2* variant decreased to 61.4% (Figure 4B).

### Transcriptional profiling identifies dysregulated metabolism-related, immune-related and neurophysiological-related signaling pathways resulting from compound heterozygous variations c.854A>G and c.1183g>T in *ACO2*

**Figure 5:**
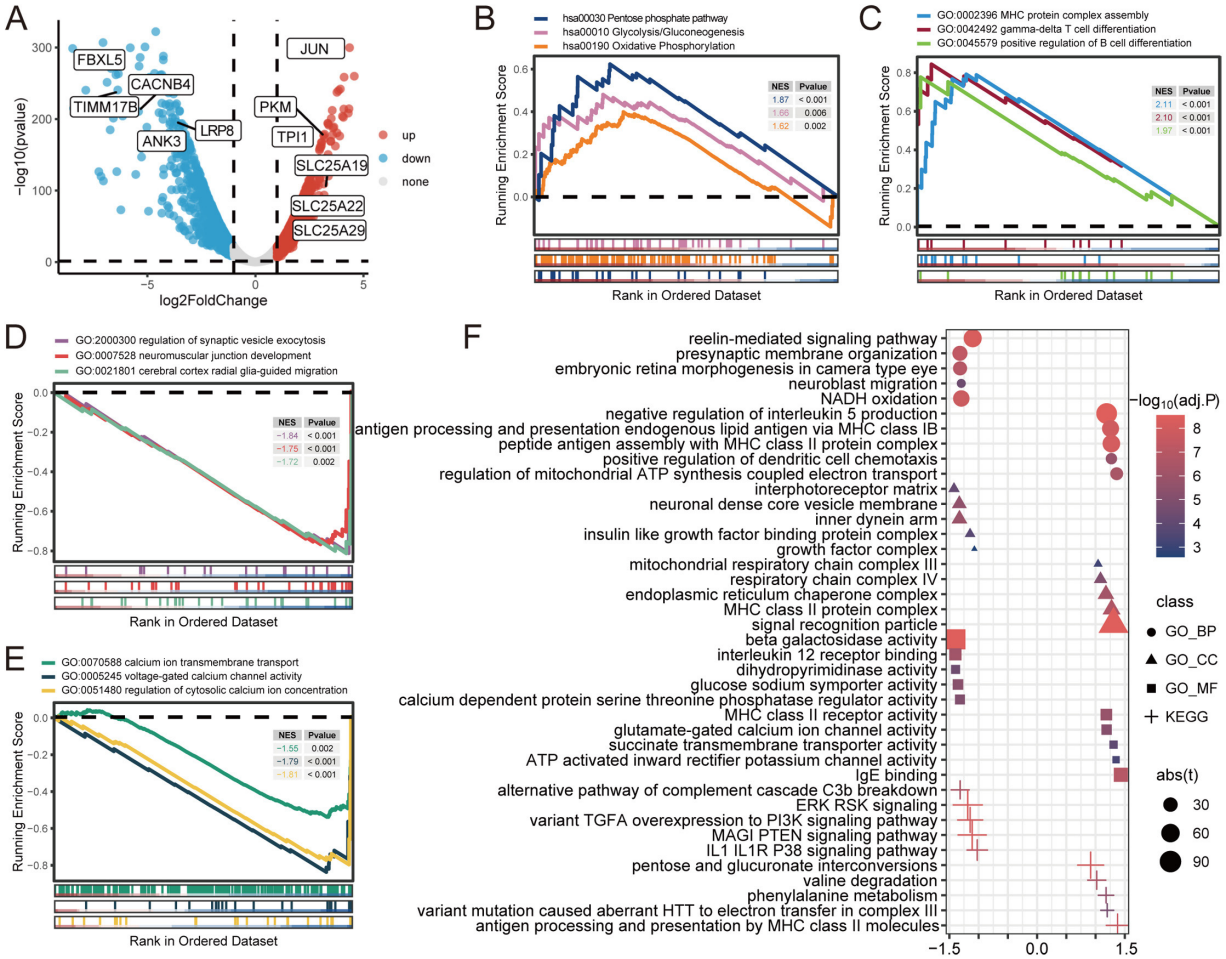
Gene differential analysis and gene sets enrichment analysis. **(A)** Volcano plot showing the differentially expressed genes. **(B)** GSEA for indicated KEGG pathways. **(C–E)** GSEA for indicated GO terms. **(F)** GSVA for indicated GO terms and KEGG pathways.

To determine the impact of compound heterozygous variants c.854A>G (p.Asn285Ser) and c.1183C>T (p.Arg395Cys) in *ACO2* on transcriptome-wide variation, RNA-seq analysis was performed on peripheral blood leukocytes obtained from family members I-2, II-1, and II-2. According to the established threshold, there were 1,082 significantly upregulated and 1,279 significantly downregulated differentially expressed genes in biallelic variant cells compared with monoallelic variant cells (Figure 5A). GSEA results showed that biallelic variant cells were positively correlated with the Pentose Phosphate Pathway (KEGG:hsa00030, *P* < 0.001), Glycolysis/Gluconeogenesis (KEGG:hsa00010, *P* = 0.006), and Oxidative Phosphorylation (KEGG:hsa00190, *P* = 0.002), as well as MHC Protein Complex Assembly (GO:0002396, *P* < 0.001), Gamma-Delta T Cell Differentiation (GO:0042492, *P* < 0.001), and Positive Regulation of B Cell Differentiation (GO:0045579, *P* < 0.001; Figures 5B, C). Conversely, they were negatively correlated with the Regulation of Synaptic Vesicle Exocytosis (GO:2000300, *P* < 0.001), Neuromuscular Junction Development (GO:0007528, *P* < 0.001), Cerebral Cortex Radial Glia-Guided Migration (GO:0021801, *P* = 0.002), Regulation of Cytosolic Calcium Ion Concentration (GO:0051480, *P* < 0.001), Calcium Ion Transmembrane Transport (GO:0070588, *P* = 0.002), and Voltage-Gated Calcium Channel Activity (GO:0005245, *P* < 0.001; Figures 5D, E). The GSVA results showed that metabolism-related, immune-related, neurophysiological-related, and calcium-related signaling pathways were differentially enriched in biallelic and monoallelic variant cells (Figure 5F). These transcriptome analyses reveal genes related to neurophysiological functions (GO:2000300, GO:0007528, GO:0021801), which can serve as candidate key molecules for *ACO2* deficiency-induced neuronal degeneration (Table 2).

**Table 2:** Potential candidate genes contributing to the ACO2 deficiency-induced neuronal degeneration.

**GO terms**	**Description**	**Genes**
GO:2000300	Regulation of synaptic vesicle exocytosis	*LRRK2/DVL1/PFN2/CACNB4/SLC4A8/SEPTIN5*
GO:0007528	Neuromuscular junction development	*CACNB3/UNC13B/KY/NEDD4/SPG11/LRRK2/ERBB2/ANK3/DVL1/MYCBP2/AGRN/CACNB4/ALS2*
GO:0021801	Cerebral cortex radial glia-guided migration	*BMERB1/LRP8/SYNE2/ADGRG1*
GO:0021516	Dorsal spinal cord development	*PBX3/ASCL1/GDF7/DRAXIN/DRGX/WNT3A/UNCX/GSX1/HOXB8/LMO4/LHX3/GDNF/PROX1/ LHX1/MDGA1*
GO:0008089	Anterograde axonal transport	*ARL8A/TERF2/HSPB1/AP3S2/SOD1/DTNBP1/SPG7/SNAPIN/BLOC1S5/BLOC1S4/NEFL/KIF3A/AP3B2/BLOC1S6/AGBL4/KIF3B/BLOC1S1/DLG2/CNIH2/MAP2/ARL8B/MAPK8IP3/BLOC1S2/AP3M2*
GO:0120111	Neuron projection cytoplasm	*ARL8A/BAIAP2/BLOC1S1/BLOC1S5/DST/FLOT2/GRIK3/HNRNPU/KIF4A/KIF5A/KIF5B/PQBP1/RAB27B/RANGAP1/SPAST/STAU1/UHMK1/AP3D1/BLOC1S2/FMR1/KIF4A/KIF5C/NDEL1/NEFL/TMEM108/WASF1*
GO:0008088	axo-dendritic transport	*ARL8A/BLOC1S1/BLOC1S5/BORCS5/DST/FLOT2/HNRNPU/KIF4A/KIF5A/KIF5B/RAB27B/SPAST/STAU1/AP3D1/BLOC1S2/KIF5C/NDEL1/NEFL/TMEM108/WASF1*

### Identification of the potential molecular mechanism of *ACO2* in ICRD

**Figure 6:**
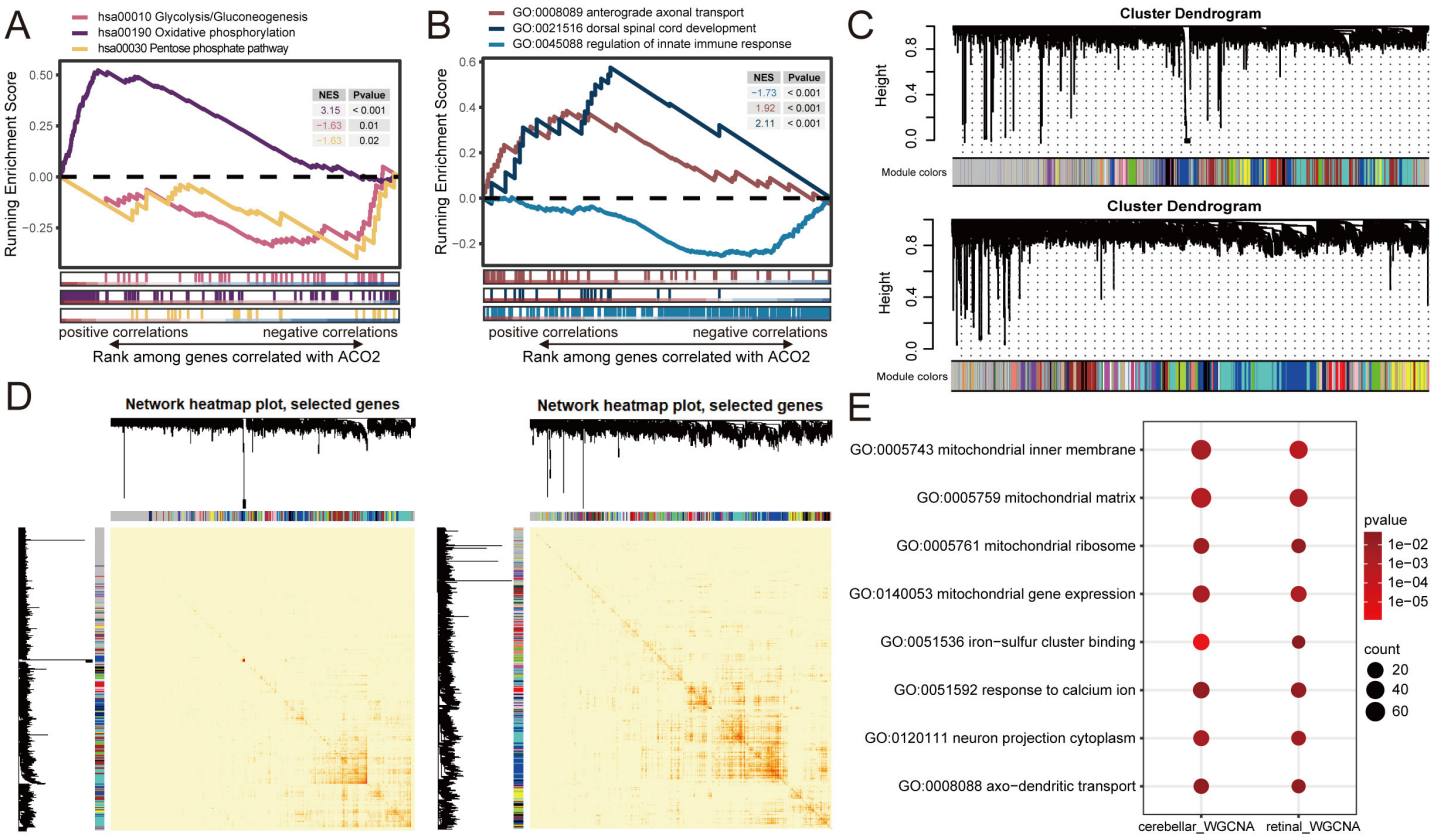
Identification of ACO2-related enrichment signatures in cerebellar and retinal organoids from GEO transcriptomic datasets. **(A, B)** Running enrichment scores by GSEA for indicated gene sets significantly associated with *ACO2* expression by Pearson's correlation across GSE161549 and GSE254830 datasets. **(C)** Hierarchical cluster dendrogram showing co-expression modules identified using WGCNA. Branches with different colors correspond to 19 and 30 co-expression modules from GSE161549 (upper) and GSE254830 (lower) datasets, respectively. **(D)** Network heatmap plots of co-expression modules in GSE161549 **(left)** and GSE254830 **(right)** datasets. **(E)** Shared enrichment signatures of co-expressed genes with *ACO2* in GSE161549 and GSE254830 datasets.

We utilized transcriptome data of cerebellar and retinal organoids retrieved from the GEO database (GSE161549 and GSE254830) to elucidate the potential molecular mechanism of *ACO2* in the development of ICRD. Both datasets were integrated by ranking all 15,364 expressed genes based on the Pearson correlation of each gene with *ACO2* expression. The ranked gene list was then used to perform a single-gene GSEA. The GSEA results revealed that *ACO2* expression was positively associated with Oxidative Phosphorylation (KEGG:hsa00190, *P* < 0.001), Dorsal Spinal Cord Development (GO:0021516, *P* < 0.001), and Anterograde Axonal Transport (GO:0008089, *P* < 0.001), and negatively associated with the Pentose Phosphate Pathway (KEGG:hsa00030, *P* = 0.02), Glycolysis/Gluconeogenesis (KEGG:hsa00010, *P* = 0.01), and Regulation of Innate Immune Response (GO:0045088, *P* < 0.001; Figures 6A, B). These data support our findings that *ACO2* dysfunction leads to abnormal metabolism-related, immune-related, and neurophysiological-related (GO:0021516, GO:0008089) signaling pathways (Figures 5B–D; Table 2).

We next performed WGCNA to identify co-expression patterns of genes in cerebellar and retinal organoids. WGCNA analysis of the GSE161549 and GSE254830 datasets identified 19 and 30 co-expression modules, respectively. For the genes co-expressed with *ACO2*, 2,039 genes in the blue module were identified from the GSE161549 dataset of cerebellar organoid, and 969 genes in the brown module were identified from the GSE254830 dataset of retinal organoid (Figure 6C). GO enrichment analysis of the two groups of co-expressed genes revealed shared enrichment for signatures involved in mitochondrial substructure and gene expression, iron-sulfur cluster binding, response to calcium ions, neuron projection cytoplasm (GO:0120111), and axo-dendritic transport (GO:0008088; Figure 6E). These results further identify the potential molecular networks of *ACO2* in cerebellar and retinal development (GO:0120111, GO:0008088; Table 2).

**Figure 7:**
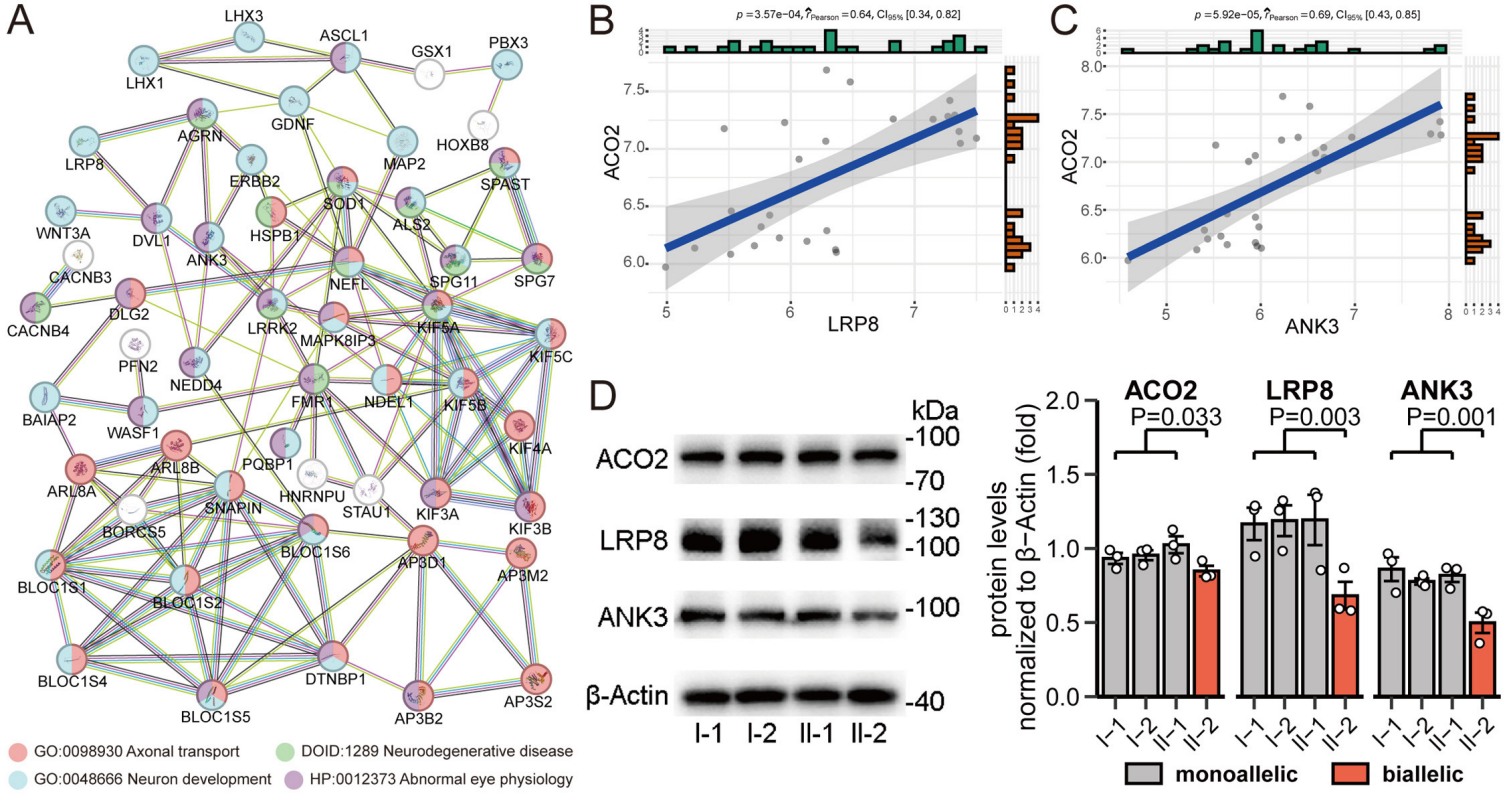
Identification and validation of key molecules in neurophysiological features. **(A)** STRING network of selected human proteins with GO annotation. **(B)** Positive correlation between *ACO2* and *LRP8* expression in integrated GSE161549 and GSE254830 datasets. **(C)** Positive correlation between *ACO2* and *ANK3* expression in integrated GSE161549 and GSE254830 datasets. **(D)** Western blotting of leukocytes from four family members showing LRP8 and ANK3 protein levels. β-Actin was used as a loading control **(left)**. Data are represented as the mean ± SD. Statistical significance between groups was determined using unpaired *t*-test **(right)**.

To better understand the biological signatures underlying the association between *ACO2* variations and neural traits, we conducted protein-protein interaction network analysis on all molecules mapped to neurophysiological features. Analysis of functional protein-protein interactions among the 80 human proteins was conducted using the STRING online database, revealing highly interconnected networks represented by proteins involved in Axonal Transport (GO:0098930), Neuron Development (GO:0048666), Neurodegenerative Disease (DOID:1289), and Abnormal Eye Physiology (HP:0012373; Figure 7A). Among them, *LRP8* and *ANK3*, which have broad tissue expression characteristics, were identified as downregulated differentially expressed genes in biallelic variant cells and showed a positive correlation with *ACO2* expression levels in the integrated GSE161549 and GSE254830 datasets (Figures 7B, C). We verified that ACO2, LRP8, and ANK3 protein levels were decreased in *ACO2* biallelic variant cells by western blotting, suggesting that LRP8 and ANK3 are involved in the neurologic phenotypes of ICRD caused by *ACO2* mutations (Figure 7D).

## Discussion

Previous pedigree-based studies have established that pathogenic variants in *ACO2* are associated with a diverse range of clinical manifestations, including ICRD, spastic paraplegia, and optic atrophy (Neumann et al., 2020; Cerrada et al., 2019; Charif et al., 2021; Sharkia et al., 2019; Spiegel et al., 2012; Blackburn et al., 2020; Abela et al., 2017; Metodiev et al., 2014; Bouwkamp et al., 2018; Tozawa et al., 2021; Marelli et al., 2018; Gibson et al., 2020). In this study, we identified novel compound heterozygous pathogenic variants in *ACO2* [c.854A>G (p.Asn285Ser) and c.1183C>T (p.Arg395Cys)] in an individual from a Chinese family affected by ICRD. This condition was characterized by psychomotor retardation, truncal hypotonia, ophthalmologic abnormalities, cerebellar atrophy, and optic nerve damage. To contribute to our understanding of *ACO2* variants and their associated phenotypes, we have compiled a comprehensive review of reported genotypes and phenotypes (Figure 2A). While this analysis has revealed potential hotspots for pathogenic variants, additional reports are necessary to definitively confirm the genotype-phenotype correlation of the two rare variants described in this case. The pathogenicity of *ACO2* variants was supported by bioinformatics predictions, as well as a significant decrease in mitochondrial aconitase activity, mtDNA copy number, and ACO2 protein levels, which are consistent with previous findings (Neumann et al., 2020; Lail et al., 2023; Metodiev et al., 2014; Ricci et al., 2024). This was accompanied by significantly enriched gene sets of metabolism-related, immune-related, neurophysiological-related, and calcium-related signaling pathways revealed by transcriptome data. These results suggest that, in addition to mitochondrial dysfunction caused by *ACO2* functional defects, immune and neurophysiological functions are also involved. The pleiotropic effects of *ACO2* are reflected in mitochondria, the key organelles for cellular energy, and mitochondrial dysfunction underlies numerous diseases (Donnino et al., 2011). In support, we further validated the potential functions of *ACO2* co-expressed genes in these pathways using transcriptome profiles of cerebellar and retinal organoids (GSE161549 and GSE254830), reflecting the applicability of transcriptome data obtained from leukocytes to explore how *ACO2* deficiency affects the neurophysiology of ICRD. Our findings suggest a role for *ACO2* in mitochondrial function, establish that defective ACO2 function underlies multiple abnormal gene sets, and provide additional pathophysiological insights into the relationship between mitochondrial dysfunction and neural atrophy.

ICRD provides a robust model for the phenotype of mitochondrial diseases and neural atrophy, which may help reveal the pathophysiology of mitochondrial diseases with multiple genotypes or other diseases manifested as neural atrophy. The precise mechanisms by which *ACO2* affects neuroatrophy are ill-defined, and several mechanisms might be involved.

Mitochondrial ACO2 is a key enzyme in the canonical TCA cycle, and the extent of damage to its activity caused by pathogenic mutations is closely related to the heterogeneity of disease phenotypes (Blackburn et al., 2020; Metodiev et al., 2014). Previous literatures have revealed that pathogenic *ACO2* mutations are associated with reduced enzyme activity, abnormal expression levels of mitochondrial respiratory chain enzymes, impaired mitochondrial respiratory function, and decreased mitochondrial DNA levels (Sadat et al., 2016; Spiegel et al., 2012; Metodiev et al., 2014; Ricci et al., 2024). However, the mechanism by which ACO2 abnormalities lead to ICRD neuropathies following mitochondrial metabolic disorders has not been elucidated. Mitochondrial dysfunction or defects in the mitochondrial respiratory chain can lead to neurodegenerative diseases, involving molecular mechanisms such as oxidative stress, proteasome, and autophagy (Zhu et al., 2023; Lin and Beal, 2006; Ghavami et al., 2014; Nithianandam et al., 2024). In this study, we demonstrated that biallelic *ACO2* variants decreased mitochondrial aconitase activity and mtDNA levels. Our enrichment analysis using multiple datasets revealed a positive correlation between *ACO2* and the Oxidative Phosphorylation (KEGG:hsa00190) gene set, as well as a negative correlation between *ACO2* and the Pentose Phosphate Pathway (KEGG:hsa00030) and Glycolysis/Gluconeogenesis (KEGG:hsa00010) gene sets. We observed a positive correlation between pathogenic *ACO2* variants and Oxidative Phosphorylation (KEGG:hsa00190), which may be explained by the compensatory activation of non-canonical oxidative metabolism. Arnold et al. observed in non-small cell lung cancer cells and embryonic stem cells that after *ACO2* disruption, cells transitioned from canonical mitochondrial TCA cycle metabolism to non-canonical TCA cycle metabolism mediated by a cascade involving the mitochondrial transporter SLC25A1, ATP citrate lyase (ACL), and malate dehydrogenase (MDH) (Arnold et al., 2022). The potential advantages of the latter include retaining reduced carbon, regenerating cytosolic NAD+ required for glycolysis, maintaining oxaloacetate regeneration, and minimizing mitochondrial NADH production (Arnold et al., 2022). We identified upregulated mitochondrial transporter family members *SLC25A19, SLC25A22*, and *SLC25A29*, which are involved in the transport of thiamine pyrophosphate, glutamate, and ornithine, respectively. These may contribute to the compensatory maintenance of mitochondrial homeostasis under *ACO2* disruption. In the context of this metabolic shift, the oxidative stress, proteasome, or autophagy pathways previously thought to be associated with neurodegenerative diseases have not been significantly enriched, and the pathophysiological mechanisms of ACO2-related neuroatrophy remain to be determined.

*ACO2* is also positively correlated with immune-related pathways. This is not surprising, given recent evidence indicating that *ACO2* suppresses immunity by modulating oxaloacetate and the mitochondrial unfolded protein response (Kim et al., 2023). Although abnormal immune levels are a potential cause of neurodegeneration, this study observed no abnormalities in peripheral blood immune cell counts in the proband, and previous research did not describe immune-related phenotypes (Spiegel et al., 2012; Blackburn et al., 2020). This suggests that the neuropathy of ICRD may not be closely related to abnormal immune levels, and thus, we cannot comment on the possible relationship between ACO2-related neuroatrophy and immunity.

Other pathways are also involved in neuropathy, such as those regulating calcium ions. Dysfunction in this pathway can result in neuronal cell death (Calvo-Rodriguez and Bacskai, 2021). We identified downregulated *CACNB4* as a candidate key gene from differentially expressed genes, calcium-related, and neurophysiological-related signaling pathways. However, there was no significant correlation between the expression levels of *CACNB4* and *ACO2* in cerebellar and retinal organoids. Therefore, we speculate that the altered calcium-related pathway provides some indirect support for ACO2-related neuropathy.

In addition, to delineate how downstream molecules of *ACO2* deficiency lead to clinical phenotypes, we focused primarily on neurophysiologically related phenotypic features, based on the hypothesis that these would best reflect the pathophysiology at stake. We identified genes involved in neurophysiological functions as key candidates, constructed a PPI interaction network, and identified key mediating molecules involved in *ACO2* mutations causing neuropathies. Functional annotation based on the PPI network revealed the association between these genes and abnormal eye physiology, which may explain the ophthalmic abnormalities in ICRD patients. Due to their neurophysiological functions, downregulated differentially expressed genes, and positive correlation with *ACO2* expression, *ANK3* and *LRP8* were considered promising key candidate genes. Among them, *ANK3* plays an important role in coordinating appropriate action potential initiation and axonal propagation (Smith and Penzes, 2018), and genetic variations in the *ANK3* gene are associated with human neurodevelopmental disorders (Kloth et al., 2021; Fang et al., 2023). Piguel NH et al. reported a decrease in dendritic complexity and the number of dendritic spines in a conditional knockout mouse model lacking *Ank3* expression in adult forebrain pyramidal neurons (Piguel et al., 2023). In our WGCNA analysis of cerebellar and retinal organoids, we identified *ACO2* co-expressed genes associated with axo-dendritic transport (GO:0008088; Figure 6E), suggesting a degree of functional similarity between *ACO2* and *ANK3*. A similar functional relationship has also been observed between *ACO2* and *LRP8*. *LRP8* plays a crucial role in neuronal migration, polarization, and differentiation during neuronal development by mediating Reelin signaling (Telese et al., 2015; Santana and Marzolo, 2017). Our GSVA analysis revealed a significant downregulation of Reelin-mediated signaling in cells with biallelic *ACO2* variants (Figure 5F). The protein expression levels of ANK3 and LRP8 were verified by western blot analysis. Although the expression levels of these proteins were only evaluated in leukocytes, considering the ubiquitous expression characteristics of these two proteins, and the previous successful cases of identifying neurogenetic diseases using transcriptome data from leukocyte samples (Rentas et al., 2020), it suggests the possibility that these two proteins may serve as key mediator molecules in the development of neural atrophy caused by *ACO2* mutations.

In summary, our study identified compound heterozygous variations that expand the spectrum of pathogenic *ACO2* variants and revealed key mediating molecules, *LRP8* and *ANK3*, between *ACO2* mutations and the development of neuropathy. These results provide in-depth support for the pathogenicity of *ACO2* genetic variations, important references for genetic counseling, and significant pathophysiological insights into neuropathy caused by *ACO2* variations and related mitochondrial dysfunction.

## Data Availability

The datasets presented in this study can be found in online repositories. The names of the repository/repositories and accession number(s) can be found in the article/supplementary material.
